# Understanding memory dynamics in stroke patients: Learning and forgetting patterns based on verbal recall

**DOI:** 10.1111/jnp.70004

**Published:** 2025-07-15

**Authors:** Selma Lugtmeijer, Edward H. F. de Haan, Roy P. C. Kessels

**Affiliations:** ^1^ Centre for Human Brain Health and Institute for Mental Health, School of Psychology University of Birmingham Birmingham UK; ^2^ Donders Institute for Brain, Cognition and Behaviour Radboud University Nijmegen The Netherlands; ^3^ Department of Psychology University of Amsterdam Amsterdam The Netherlands; ^4^ St Hugh's College University of Oxford Oxford UK; ^5^ Psychology Department University of Nottingham Nottingham UK; ^6^ Vincent van Gogh Institute for Psychiatry Venray The Netherlands; ^7^ Radboudumc Alzheimer Center Radboud University Medical Center Nijmegen The Netherlands

**Keywords:** accelerated long‐term forgetting, episodic memory, forgetting, learning, long‐term memory, stroke, verbal memory

## Abstract

Memory deficits are common post stroke. Most episodic memory tests consist of a learning phase, immediate recall and a 30‐min delayed recall test. Recent research suggests a proportion of stroke patients exhibit accelerated long‐term forgetting after a longer delay. Based on a word‐list learning test in stroke patients and controls, we demonstrate that stroke patients recalled fewer words after five learning trials, and on 30‐min and 1‐week delayed recall tests, caused by shallower learning curves and higher percentages of forgetting.

## INTRODUCTION

Memory deficits are common after stroke, but their presentation is heterogeneous (O'Sullivan et al., 2023). Given their high prevalence, memory tests are a crucial component of post‐stroke neuropsychological assessment. Most clinically used episodic memory tests consist of a learning phase followed by an immediate recall test and a delayed recall test after approximately 30 min. However, many stroke patients may exhibit abnormal forgetting over a prolonged interval, even in the absence of short‐delay memory impairment. This suggests that assessing longer‐term forgetting rates could assist in identifying patients with memory decrements that may otherwise go undetected (Lammers, Lugtmeijer, et al., 2022).

Forgetting over a prolonged interval or accelerated long‐term forgetting (ALF) is defined as abnormal forgetting over hours to weeks of information that is typically encoded and retained over a short interval (Butler et al., 2019; Elliott et al., 2014). To assess ALF, a third measurement—days or weeks after initial encoding—is added to the test procedure. While ALF is a characteristic of patients with temporal lobe epilepsy (Elliott et al., 2014), evidence for ALF in other brain‐injured patient groups is less consistent (Geurts et al., 2015). In one study of minor stroke and patients with transient ischaemic attack (TIA), recognition and recall were measured using a word‐list learning paradigm, with a 30‐min and 1‐week delayed test. Patients performed worse than stroke‐free controls only in the 1‐week delayed recall test, but not in the short delay or recognition tests (Geurts et al., 2019). Lammers, Lugtmeijer, et al. (2022) did not find any overall differences in forgetting rates between stroke patients and controls in a 30‐min and 1‐week delayed visual recognition memory test. However, they were able to identify a subgroup of patients who displayed ALF.

In this study, we extend the findings of Lammers, Lugtmeijer, et al. (2022) by investigating the dynamics of verbal learning and forgetting in stroke patients compared to an age‐matched control group. To address previous limitations, we employed a free recall task and included a young control group as a benchmark to determine typical patterns of forgetting in the absence of effects of age or pathology. To assess accelerated long‐term forgetting, we supplemented a standard word‐list learning protocol with a 1‐week delayed free recall test as free recall measures are more sensitive to forgetting than recognition tests (Kopelman, 2025).

## METHODS

### Participants

Patients were included via the multicentre cohort study “A functional Architecture of the Brain for Vision” (FAB4V; Lammers, van den Berg, et al., 2022). From this prospective cohort, 81 patients completed the RAVLT protocol of whom 60 had a T2 FLAIR MRI scan. The assessment took place between 2 weeks and 2 years post‐stroke.

Two control groups were recruited, one with younger adults (*N* = 33, age range 21–49) to define normal rates of forgetting, and an age‐matched control group (*N* = 127, age range 50–87). Participants gave written informed consent.

### Materials and analysis

The word‐list learning paradigm was based on the Dutch version of the Rey Auditory Verbal Learning Test (RAVLT) (Van Der Elst et al., 2005), which comprises 15 unrelated words read aloud to participants during each of five learning trials. The learning outcome was defined as the score on trial five (trial V). After a 30‐min delay, participants completed a delayed recall test (short delay, SD). Additionally, a surprise long‐term delayed recall test (long delay, LD) was administered between 5 and 15 days via phone call.^1^ No interference list was included in this paradigm.

Learning ratio was calculated as the percentage increase from trial I to trial V (Spencer et al., 2023), taking into account the number of words in the list (15):

1. Learning ratio = ((score trial V − score trial I)/(list length − score trial I))

Forgetting scores, relative to initial acquisition (trial V), were computed to quantify the number of forgotten items as a percentage of items recalled at trial V (Lammers, Lugtmeijer, et al., 2022):

2 T2 forgetting rate = ((score trial V − score SD)/score trial V) × 100
3 T3 forgetting rate = ((score trial V − score LD)/score trial V) × 100

Forgetting rates that were negative were set to zero (less than 5% of participants), as a better score on the recall than the initial learning indicates no forgetting (Lammers, Lugtmeijer, et al., 2022).

To examine group differences, we conducted six separate linear regression models (lm() in R; on raw scores: trial V, SD and LD scores, and derived scores for learning ratio, and T2 and T3 forgetting rates). The model included group as a predictor and age and sex as covariates. In case of violation of the assumptions, robust regression (rlm()) and robust standard errors (rSE) were used. To control for multiple comparisons, we treated raw performance scores and derived measures as separate families of tests and applied Bonferroni correction (adjusted *α* = .05/3 = .017).

To further assess different patterns of forgetting, patients and age‐matched controls were split into subgroups. Prevalence of rapid forgetting (RF; abnormal forgetting over a short delay; RF) and ALF was determined based on the performance of the young control group. Prevalence of RF and ALF was determined based on the performance of the young control group. RF was defined as a T2 forgetting rate above 2 standard deviations of the mean of the young control group (a higher forgetting rate indicates worse performance). A participant was labelled as showing ALF when they scored within 2 standard deviations on the T2 forgetting rate and had a T3 forgetting rate of more than 2 standard deviations above the mean of the T3 forgetting rate of the young control group. Participants who fell in neither group were labelled as normal forgetting (NF). A lesion overlap map was plotted for each stroke patient memory subgroup for descriptive reasons. Due to small numbers, no formal lesion‐symptom analyses were conducted.

## RESULTS

### Effects of group on memory performance

**FIGURE 1:**
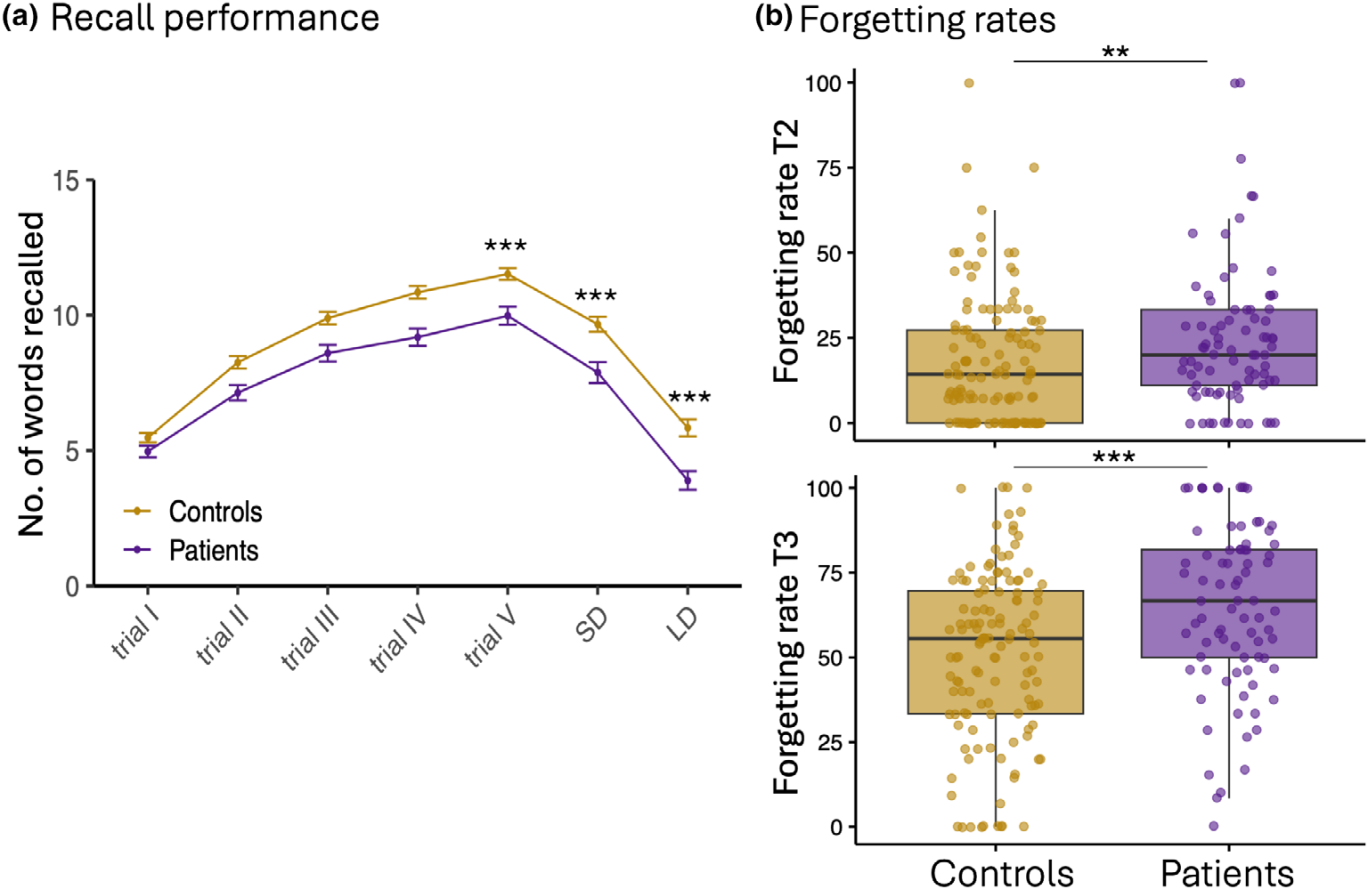
Raw memory performance split by group. (a) Performance of stroke patients and age‐matched controls at trials I–V, SD and LD. (b) T2 and T3 forgetting rates. The box plot shows the median (50th percentile), interquartile range (IQR; 25th–75th percentile) and the 1.5 × IQR (whiskers). Significant difference between the groups, <.001***, <.01**.

There were no significant differences in sex distribution between young controls, age‐matched controls and patients (*χ* ^2^(2) = 3.74, *p* = .15). The stroke and age‐matched control group were successfully age‐matched (*M* age patients 63.1 and controls 64.2; *t* = −.86, *p* = .341). Figure 1a shows the raw overall memory performance for the patient and age‐matched controls for all time points, including the learning trials. Linear regression indicated that recall performance was significantly lower in patients than in controls across all three time points (trial V: *β* = −1.68, rSE = .37, *p* < .001; SD: *β* = −2.00, SE = .42, *p* < .001; LD: *β* = −2.21, rSE = .41, *p* < .001, respectively, trial V, 30‐min and 1‐week delay, corrected for age and sex). The model explained approximately 24% of the variance in recall performance ((robust) *R* ^2^ = .24, .24 and .24).

The learning ratio was lower in patients than in age‐matched controls (*β* = −13.10, SE = 3.31, *p* < .001, *R* ^2^ = .16). Forgetting rates, measured as the percentage decline from initial learning, were significantly higher in patients compared to controls for the 30‐min delay (T2, *β* = 6.67, rSE = 2.31, *p* < .01, robust *R* ^2^ = .09) and, to a greater extent, for the 1‐week delay (T3, *β* = 16.35, rSE = 3.32, *p* < .001, robust *R* ^2^ = .18; Figure 1b).

### Different memory profiles

**FIGURE 2:**
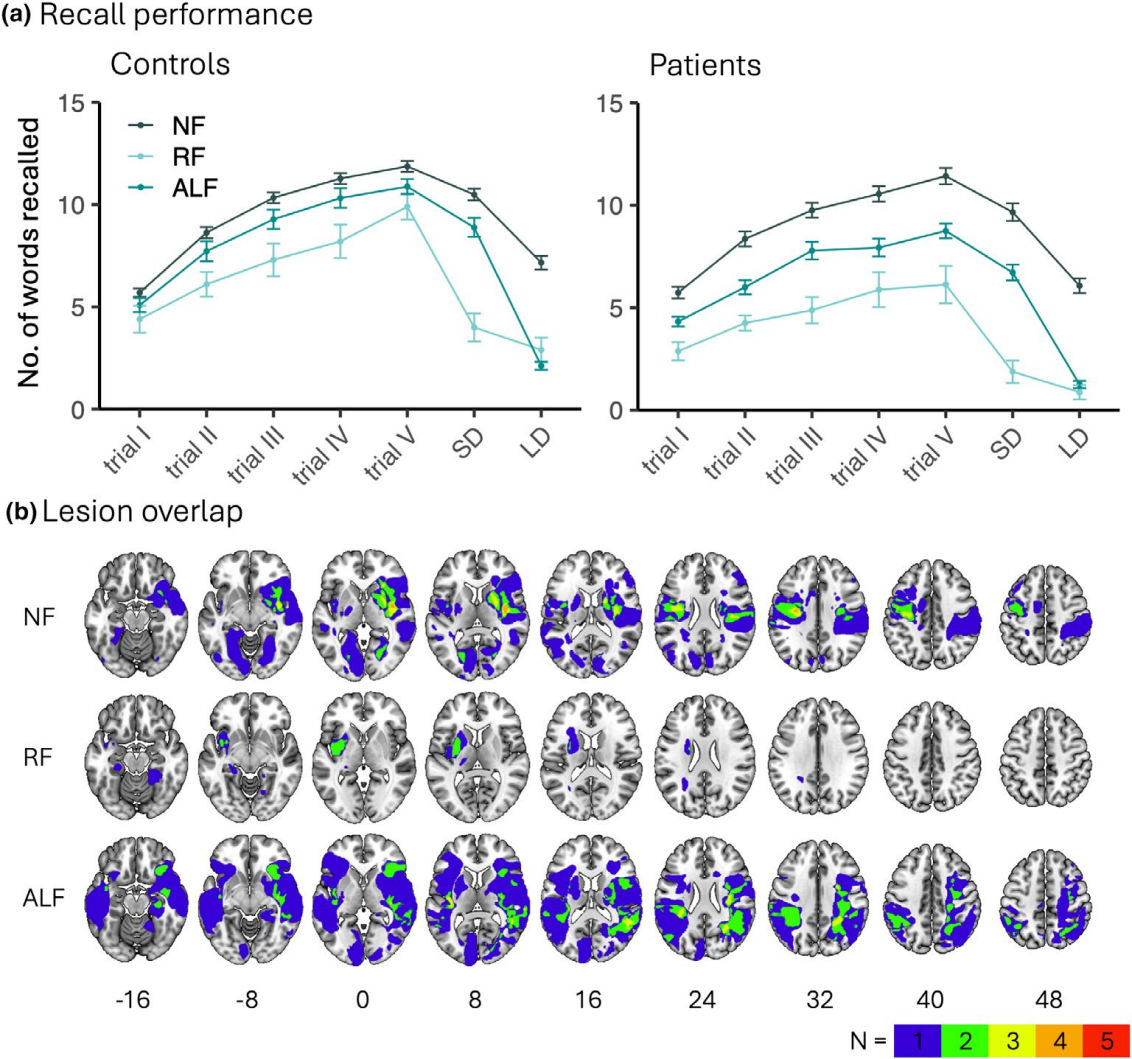
Memory subgroup classification of participants. (a) Performance on the RAVLT, five learning trials and two measures for delayed recall (SD and LD). In the left panel scores of age‐matched controls (Normal forgetting [NF] *N* = 92, Rapid forgetting [RF] *N* = 10, Accelerated long‐term forgetting [ALF] *N* = 25), in the right panel performance of stroke patients (NF *N* = 45, RF *N* = 8, ALF *N* = 28). The error bars in the plots represent the Standard Errors of the Mean. (b) Lesion overlap per memory subgroup. Maximum overlap is 5. No statistical analyses were conducted on the overlap because of the small sample size of the subgroups. Numbers indicate MNI coordinates. The left hemisphere is depicted on the left.

Figure 2a shows the raw RAVLT scores for patients and age‐matched controls per subgroup. No further statistics were conducted on the differences in performance between the subgroups as the subgroups were defined based on their performance. Figure 2b shows the overlap of lesions for patients in each of the memory subgroups. The lesion overlap within both the RF and ALF stroke patient groups is minimal (<3 patients), suggesting a lack of a strong lesion‐symptom association.

Table 1 presents demographics and derived RAVLT scores. A chi‐squared test revealed a significant difference in the distribution of the three memory subgroups between patient and age‐matched control groups (*χ* ^*2*^(2) = 6.67 *p* = .036). Specifically in the patient group the proportion of the ALF classifications was much higher than in the age‐matched control group (34.6% vs. 19.7%), while the percentage of participants with RF was comparable (9.9% vs. 7.9%). A one‐way ANOVA showed significant age differences across the six groups (three memory subgroups in both patients and age‐matched controls). Specifically, a Games—Howell post‐hoc test showed that RF patients were older than other patients and the NF controls; none of the other pairwise group comparisons were significant. A Fisher's Exact Test revealed a significant overall association between sex and memory subgroup (*p* = .009). However, follow‐up pairwise comparisons (FDR‐corrected for multiple testing) indicated no significant differences in sex distribution between any subgroup pairs. There were no differences in lesion characteristics (volume hemisphere time since stroke) between the subgroups.

**TABLE 1:** Participant demographic information and stroke characteristics for each RAVLT subgroup.

	Stroke patients				Age‐matched controls			
	NF	RF	ALF	*p*‐value	NF	RF	ALF	*p*‐value
*N* (%)	45 (55.6%)	8 (9.9%)	28 (34.6%)	–	92 (72.4%)	10 (7.9%)	25 (19.7%)	.036
Age (M ± SD)	59.96 (11.50)	73.00 (4.63)	65.21 (8.85)	–	53.68 (17.25)	61.73 (16.34)	65.75 (8.45)	.002
Sex (M:F)	27:18	8:0	22:6	–	50:42	4:6	19:6	.009
Education (Median, range)	6 [2–7]	5 [2–7]	5 [2–7]	–	6 [3–7]	5 [4–7]	6 [3–7]	.118
Stroke characteristics								
Time since stroke in days (Median, range)	46 [17–124]	38 [26–101]	64 [23–230]	.228	–	–	–	–
Lesion volume in cm^3^ (Median, range)	4.85 [.07–85.12]	6.97 [1.26–11.88]	12.72 [.002–136.89]	.424	–	–	–	–
Hemisphere (L:R:B)	16:12:7	4:0:1	7:10:3	.303	–	–	–	–
RAVLT								
Learning ratio (M ± SD)	63.62 (24.25)	26.91 (20.04)	41.42 (16.51)	–	70.85 (24.39)	54.39 (19.33)	57.94 (17.62)	–
T2 forgetting rate (M ± SD)	15.95 (11.97)	72.78 (18.28)	23.92 (11.94)	–	10.28 (15.02)	63.24 (16.78)	19.75 (14.91)	–
T3 forgetting rate (M ± SD)	46.81 (17.45)	89.58 (11.28)	86.61 (9.27)	–	37.49 (20.72)	66.62 (24.19)	80.42 (8.57)	–

*Note*: Education level is expressed as seven categories, based on the Dutch educational system that is comparable with the International Standard Classification of Education.

## DISCUSSION

This study investigated learning and forgetting patterns in stroke patients. Stroke patients recalled fewer words than age‐matched controls after trial V, and 30‐min and 1‐week delayed recall intervals and showed reduced word learning across repeated trials. Forgetting rates (percentage of forgotten words) were slightly higher in patients than age‐matched controls over the short delay, with a more pronounced difference over the longer delay. These different forgetting patterns reflect the notably higher prevalence of ALF in patients compared to controls, without a higher prevalence of RF.

Learning rates were lower in stroke patients than age‐matched controls, highlighting the importance of accounting for initial learning when assessing forgetting, either through matching on initial learning (Elliott et al., 2014; Kopelman, 2025), or as in the present study, by calculating forgetting relative to the number of items initially learned (e.g., Geurts et al., 2019; Lammers, Lugtmeijer, et al., 2022). In contrast to the Lammers et al. findings, we found higher forgetting rates in patients after both delays. Key differences between these studies are the stimuli type (visual vs. verbal) and the memory test used (recognition vs. free recall). While evidence for material‐specific ALF is limited (Elliott et al., 2014), free recall measures may be more sensitive to forgetting than recognition tests (Kopelman, 2025).

Results revealed a high prevalence of ALF in stroke patients, building on previous findings of higher ALF prevalence after minor stroke or TIA compared to controls (Geurts et al., 2019) and after stroke (Lammers, Lugtmeijer, et al., 2022). Extending Lammers, Lugtmeijer, et al. (2022), we compared the prevalence of different memory profiles in stroke patients to that of age‐matched controls and showed no difference in the prevalence of RF. In both groups, a considerable proportion of participants exhibited ALF, but there was a significantly higher prevalence of ALF in stroke patients.

A long‐standing debate in the literature concerns whether ALF is quantitatively or qualitatively different from normal forgetting (Butler et al., 2019; Cassel & Kopelman, 2019; Mayes et al., 2019). It has been suggested that ALF may not represent a distinct construct but instead reflects a pattern of forgetting already present at short delays, with the only difference being a more progressive deterioration over time. This distinction could not be addressed in the current study. However, our findings show that while subtle impairments at short delays may go undetected on standard clinical tests, more pronounced differences in forgetting can be revealed by incorporating a prolonged delayed recall test.

A limitation of the current design is the presence of a floor effect at the 1‐week delay for individuals with significant episodic memory impairments. This likely reflects the use of a 15‐item word‐list learning task. However, using a free‐recall memory tasks with more stimuli will probably be too difficult, especially for older people. Still, it remains uncertain whether patients with RF and those with ALF ultimately exhibit comparable levels of forgetting after extended delays, or whether the ALF group is disproportionately impaired.

In conclusion, stroke patients, as a group, have a shallower learning curve, resulting in lower learning outcomes. They also forget a higher percentage of learned material than age‐matched controls over a short and prolonged delay, with these two measures identifying different patterns of abnormal forgetting. This suggests that the standard 30‐min delay interval used in clinical practice may be too short to identify all patients with episodic memory impairment. We demonstrate the feasibility of a 1‐week phone follow‐up with patients, which allows for identification of patients with forgetting over a prolonged delay, which would be looked over in standard clinical care.

## AUTHOR CONTRIBUTIONS

**Selma Lugtmeijer:** Conceptualization; investigation; writing – original draft; visualization; project administration; methodology; software; formal analysis; data curation. **Edward H. F. de Haan:** Conceptualization; data curation; funding acquisition; project administration; supervision; visualization; writing – review and editing. **Roy P. C. Kessels:** Conceptualization; formal analysis; methodology; project administration; resources; supervision; writing – review and editing.

## FUNDING INFORMATION

This study was supported by an ERC Advanced (Grant No. 339374) awarded to Edward H. F. de Haan.

## CONFLICT OF INTEREST STATEMENT

The authors report no competing interests.

## Data Availability

The data that support the findings of this study are openly available on Open Science Framework (OSF) at https://osf.io/5gks3/.

## Footnotes

^1^
There are some missing data for the exact interval between the learning phase and 30‐minute delayed test and the 1‐week delay. For the available data (*N* = 135), there is no significant correlation between the number of days between the short and long delay and the T3 forgetting rate (*r* = .13, *p* = 0.13).
